# A Support Vector Machine Classification of Thyroid Bioptic Specimens Using MALDI-MSI Data

**DOI:** 10.1155/2016/3791214

**Published:** 2016-05-17

**Authors:** Manuel Galli, Italo Zoppis, Gabriele De Sio, Clizia Chinello, Fabio Pagni, Fulvio Magni, Giancarlo Mauri

**Affiliations:** ^1^Department of Medicine and Surgery, University of Milano-Bicocca, Via Cadore 48, 20900 Monza Brianza, Italy; ^2^Department of Informatics, Systems and Communication, University of Milano-Bicocca, Viale Sarca 336, 20125 Milan, Italy; ^3^Department of Surgery and Translational Medicine, Section of Pathology, University of Milano-Bicocca, Milan, Italy

## Abstract

Biomarkers able to characterise and predict multifactorial diseases are still one of the most important targets for all the “omics” investigations. In this context, Matrix-Assisted Laser Desorption/Ionisation-Mass Spectrometry Imaging (MALDI-MSI) has gained considerable attention in recent years, but it also led to a huge amount of complex data to be elaborated and interpreted. For this reason, computational and* machine learning* procedures for biomarker discovery are important tools to consider, both to reduce data dimension and to provide predictive markers for specific diseases. For instance, the availability of protein and genetic markers to support thyroid lesion diagnoses would impact deeply on society due to the high presence of undetermined reports (THY3) that are generally treated as malignant patients. In this paper we show how an accurate classification of thyroid bioptic specimens can be obtained through the application of a state-of-the-art* machine learning* approach (i.e.,* Support Vector Machines*) on MALDI-MSI data, together with a particular wrapper* feature selection* algorithm (i.e.,* recursive feature elimination*). The model is able to provide an accurate discriminatory capability using only 20 out of 144 features, resulting in an increase of the model performances, reliability, and computational efficiency. Finally, tissue areas rather than average proteomic profiles are classified, highlighting potential discriminating areas of clinical interest.

## 1. Introduction

Thyroid lesion diagnosis constitutes an important issue in terms of life quality of the affected patients. Currently, this pathology is diagnosed through cytomorphological evaluation of smears obtained after an ultrasound-guided fine needle aspiration biopsy (FNAB). A category of malignancy is then assigned to specimens according to the SIAPEC-IAP (Italian Society of Anatomic Pathology and Cytology) classification [1]. In particular, a category (ranging from THY1 to THY5 in the European system) is associated with the following lesions and groups of patients: inadequate withdrawal (THY1), benign lesions (THY2), lesions with unknown malignancy potential (THY3), and malignant lesions (THY4 and THY5). The general guidelines suggest that patients diagnosed as being of THY4 or THY5, along with the ones diagnosed as being of THY3, must undergo a total thyroidectomy and a consequent lifelong hormone replacing therapy, resulting in possible complications during or after surgery and in possible compliance issues during the patient's life. Surprisingly, 70% of the THY3 cases result benign after a deep histological evaluation after surgery [1], highlighting the diagnostic problem related to the undetermined reports (THY3).

The lack of protein and genetic biomarkers to reliably support thyroid lesion diagnoses led us to exploit the discriminative power of a* machine learning* technique (i.e.,* Support Vector Machine*, SVM) applied to Matrix-Assisted Laser Desorption/Ionisation-Mass Spectrometry Imaging (MALDI-MSI) data. MALDI-MSI data has already proven itself to be capable of highlighting differences in the proteomic profile of different types of thyroid lesions [2, 3], further supporting our work. MALDI-MSI is an analytical technique that allows the study of the spatial distribution and relative abundance of a wide range of molecules directly on-tissue, without the need of any labelling or extraction processes that can possibly hinder both the molecular structure and the extraction yield of the analytes of interest [4]. For this reason MALDI-MSI has gained considerable attention in recent years and has been widely employed in several fields with successful results, from oncology and immunology to forensics and from pharmacology to the study of plants [5]. Although the advantages of MALDI-MSI are unquestionable for the explorative research, it also leads to file sizes of several gigabytes and more recently even terabytes of complex and high dimensional data from a single examined tissue slice. Computational analysis of MSI data and mining procedures are therefore challenging to be met [6].

Specifically, in this paper, we show how a* Support Vector Machine* based classification [7] can provide accurate discrimination of thyroid bioptic specimens using mass spectrometry imaging data, thus aiming at taking MALDI-MSI to the daily clinical practice to aid the clinical routine for diagnostic processes. Taking advantage of the general purpose applicability of the SVM model (broadly applied in both proteomics and more general biomolecular classification problems; see, e.g., [8] and [9], resp.) we provide accurate classification of THY3 patients to a benign or a malignant category. Moreover, to reduce the dimensionality of available data, we applied a* feature selection* algorithm (i.e.,* recursive feature elimination*; see, e.g., [10]) to a derived dataset obtained through the generation of an average (representative) spectrum per patient.

The paper is laid out as follows. In Sections 2.1 and 2.2 we briefly describe the samples and the data acquisition process. In Section 2.4 we detail the preprocessing phase. In Section 3 we report the model construction and the “standard” classification process while in Section 4 we introduce the* pixel-by-pixel classification*, important to highlight potential discriminating areas of clinical interest. We show the results in Section 6 and conclude, finally, in Section 7 by discussing our findings.

## 2. Materials and Methods

### 2.1. Patients

The study was conducted on leftover bioptic material collected at the Department of Pathology, University of Milano-Bicocca, Monza Brianza, Italy. A cohort of 43 subjects with the following characteristics (Table 1) was enrolled:

- 14 subjects diagnosed as being of THY2, 8 THY4, and 10 THY5 (for a total of 32 patients),
- 11 subjects diagnosed as being of THY3.

### 2.2. Acquisition of Mass Spectra

The cytological smears have been scanned through a ScanScope CS digital scanner (Aperio, Park Center Drive, Vista, CA, USA), to obtain a digitalised image of the specimen. After sample preparation, mass spectra were acquired using the ultrafleXtreme MALDI-TOF/TOF mass spectrometer (Bruker Daltonics GmbH, Bremen, Germany) in linear positive mode. All acquired spectra range from* m/z* 3000 to 25000, with a* raster* (namely, the spatial resolution) of 100 micrometers.

### 2.3. MALDI-MSI Data

**Figure 1:**
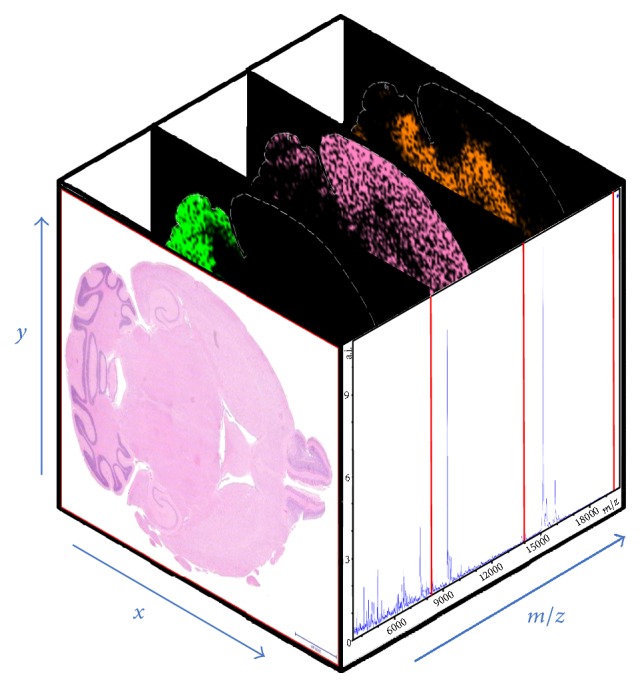
MALDI-MSI data cube. The intensity value of a specific analyte compound is localised as follows: *x*- and *y*-axis represent the spatial coordinates of the 2D digitalised tissue image (a mouse brain is shown in this example); the *z*-axis represents the* mass-to-charge* (*m/z*) ratio in the acquired spectra. For each* m/z* value in the spectrum, a 2D molecular image is computed by colouring the pixels according to the relative abundance (intensity of that* m/z* value) of the selected compound across the tissue section.

Generally, a mass spectrometry imaging dataset consists of a “data cube” (Figure 1) resulting from the acquisition of one mass spectrum for each pixel of the digitalised tissue image. By considering a particular* mass-to-charge* (*m/z*) value, we can then represent the spatial distribution of the corresponding compound (with that specific* m/z*) by colouring each pixel according to its intensity values (i.e., relative abundance) at different spatial coordinates. In other words, for each* m/z* value in the spectrum, a molecular image showing the spatial distribution of the corresponding analyte is generated, possibly highlighting regions where the selected molecule localises. Finally, spectra from specific regions of the sample can be exported and passed to the software for elaboration.

### 2.4. Data Preprocessing

Raw data provided by MALDI instruments can be viewed also as a simple collection of independent spectra which are generally unaligned and noisy. Data preprocessing is a crucial step for allowing fair comparisons and reducing both technical and analytical variability or artefacts. To provide more reliable elaboration, we first applied the following steps.

*(i) Baseline Subtraction and Smoothing*. The baseline of a spectrum is a segment connecting points with the lowest intensities on which the entire spectrum lies. The baseline is essentially made of noise (electrical noise and chemical background generated by impurities), which, in turn, hinders the feature extraction process (*peak picking*). In this work the baseline subtraction process has been computed using the* TopHat* algorithm, while the denoising was performed using the* Savitzky-Golay* smoothing, in order to bring the spectra onto the *x*-axis and to present more defined peaks (thus allowing more reproducible* peak picking* selections [11]).

*(ii) Normalisation*. Normalisation is the process that consists in the multiplication of all the intensity values in the mass spectrum by a scaling factor, which results in an intensity axis broadening or narrowing. Here we applied the so-called* total ion count* (TIC) method: all the intensities of each spectrum in the dataset are divided by the spectrum total current (i.e., the sum of all the intensities), providing each spectrum with the same integrated area under the curve [12].

*(iii) Peak Picking. *The* peak picking* was made using the* Median Absolute Deviation* (MAD) as a noise estimation method, with a* signal-to-noise* (*S/N*) ratio threshold of 3. The* peak picking* results in the selection of the highest* m/z*-intensity coordinates of the peaks in the spectra (i.e., features for the following selection phase) [13]. This leads to a massive reduction of the data dimensionality that will lead to a more computationally efficient analysis.

*(iv) Peak Alignment and Filtering*. All peaks have been aligned (with a tolerance of 2000 ppm) in order to prevent slightly analytical variations in the* m/z* values from being seen as distinct peaks. This ensures more consistent and coherent results, since possible artefacts in the identification of putative biomarkers are prevented from being generated. In addition, in order to remove false positive peaks coming from the noise, a filtering has been applied, resulting in keeping only the signals that are present in at least the 25% of all the spectra in the dataset.

The peak alignment and filtering have been performed on the entire dataset, as part of the preprocessing of the entire spectral data. Although this can potentially introduce some bias, especially in low-intensity peaks, closer to the noise and possibly not well resolved, this effect is compensated by the filtering, which was performed, for this reason, on the entire dataset. The* signal-to-noise* (*S/N*) ratio method (which we used) for* peak picking* is known to generate false positive peaks [6], and this is why the filtering is performed. Other peak picking methods, such as the orthogonal matching pursuit (OMP), which evaluates the shape of the peak rather than its intensity, are known to be more robust and reliable [6], but there are no R functions at the moment to perform peak picking with this algorithm. This could be an input for future work, to make peak picking more robust to peak shape and symmetry and to decrease the number of false positive peaks.

### 2.5. Peak-List Matrix and Data for Classification

The preprocessing step provided us with a* peak-list matrix*. This matrix with other elaborated data has been used to build and evaluate the inference model. We summarise the data we used as follows.

 
*Dataset 1: Peak-List Matrix*. As referred to above, this data is directly provided by the preprocessing step, yielding a number of aligned peaks of 144.
 
*Average Profile Data*. The obtained profiles were then used for the* average profile classification* as described in the next sections. In particular, for this task, the following two datasets were created.
 
*Dataset 2: Training Set*. It contains peak-list data from THY2, THY4, and THY5 patients.
 
*Dataset 3: Validation (Test) Set*. It contains peak-list data from THY3 patients.

While the former data was used to cross validate and obtain a classification model, the latter was applied as an external (further) validation set.

## 3. Average Profile Classification

To obtain a classifier we applied sequentially the following steps.

*(i) Recursive Feature Elimination*. In this phase, we executed a wrapper feature selection process using the training set (dataset 2) as defined previously. To avoid overfitting and allow for the classifier to work properly, we applied a repeated (2 times) 10-fold cross-validation process with the recursive feature elimination (RFE) algorithm. In particular, to evaluate the performances of the selected subsets of features, we iteratively applied a partial least squares (PLS) model (for implementation issue see R “caret” package [14]). In this way, we obtained a subset of 20 features, which, in turn, was submitted for further elaboration as described in the following step.

Feature selection decreases the risk of overfitting, especially with this reduced number of patients. When using individual spectra/pixels per patient, the risk of overfitting is reduced, but the algorithm can become slower and less efficient in terms of performances and classification capability (see the comparison of computational times in Table 2 and of classification performances in Tables 3 and 5; the process has been executed on a machine equipped with 16 GB of RAM, an Intel i7-4702mq CPU, and a 7200 rpm hard disk, on Ubuntu Linux): in fact, the mathematical formula that defines the model will be much more complicated. On the contrary, we want the algorithm to be fast and efficient, especially if an ensemble classifier is to be implemented in the future: when more algorithms are employed at the same time to vote for the class of the unknown sample, it is important that since the time taken by the process exponentially increases with the number of algorithms running, the classification is performed in reasonable time. This would also increase the translatability of the approach to the daily clinical routine. Finally, by retaining more peaks, the model can become more susceptible to variations in the peak intensity due to analytical variability and fluctuations in the instrument sensitivity and performances.

*(ii) SVM Classification*. A* Support Vector Machine* (SVM) model was trained using dataset 2 with the features provided by the* recursive feature elimination*. Moreover, the SVM was tuned to maximise the model capability, thus obtaining a classification with high performances.

A 10-fold cross-validation was performed 2 times onto the training data to assess the reliability of the SVM. The trained classifier was then tested onto validation dataset 3 (THY3 patients), returning the classification performances based upon the degree of concordance between the predicted class and the actual class, in terms of sensitivity, specificity, positive predictive value (PPV), negative predicted value (NPV), and ROC AUC (area under the curve).

## 4. Pixel-by-Pixel Classification

After testing the SVM classifier onto the average proteomic profiles, we applied the trained model to predict the class of all the individual spectra in the MALDI-MSI dataset, which is one mass spectrum for each pixel: this results in a pixel-by-pixel classification, namely, the classification of tissue areas rather than the entire proteomic profile of a patient. Since for each spectrum the physical coordinates of the digitalised image are also retained, then it is also possible to colour the corresponding pixels over the image. In other words, for each patient, a molecular image with pixels coloured according to the class is shown, highlighting differently classified tissue areas.

In the classification of new (unknown) MSI data, the algorithm preprocesses the spectra in the same way as the training dataset and aligns the peaks from the new data to the ones used for building the model. The peak filtering is performed onto the unknown MSI data before running the pixel-by-pixel classification, not in the average profile classification, in order to discard the presence of false positive peaks picked by the MAD algorithm, when individual spectra/pixels per patient are used.

## 5. Implementation

All the conceptual procedures described in this paper have been coded using the R environment (https://www.r-project.org/). The spectra were formatted as imzML files [15], imported into R using the “*MALDIquantForeign*” package [13] and processed using the “*MALDIquant*” package [13].

## 6. Results

Our primary interest was to build an accurate model able to discriminate malignant from benign thyroid bioptic specimens. Our approach was empirical: we first designed a specific knowledge discovery process (Section 3) able to provide an accurate model for case versus control classification (i.e., THY2 versus THY4 and THY5). Then we evaluated the model performances onto a validation set (THY3) as described in the previous paragraphs.

Table 5 reports the performances obtained after a repeated (2 times) 10-fold cross-validation process (using dataset 2) onto the validation set containing only patients diagnosed as being of THY3 (dataset 3). The performances are based upon the degree of concordance between the predicted class and the actual class, in terms of sensitivity, specificity, positive predictive value (PPV), negative predicted value (NPV), and ROC (*Receiver Operating Characteristic*) AUC (area under the curve).

Specifically, Table 6 displays the difference between the class that was predicted by the model and the actual class provided by the histological analysis.

**Figure 2:**
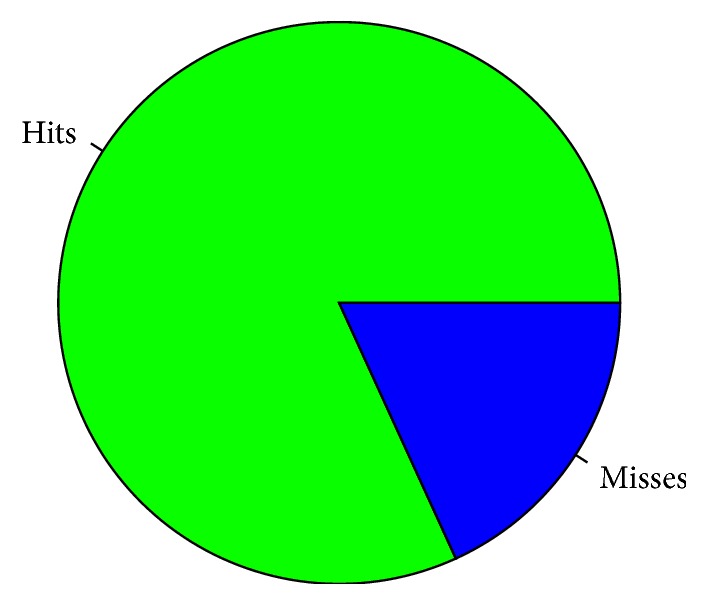
Graphical evaluation of the patient classification operated by the model. The green area is proportional to the number of correctly classified patients, while the blue area corresponds to the number of misclassifications.

A visualisation of the obtained accuracy can also be given through the pie chart in Figure 2.

**Figure 3:**
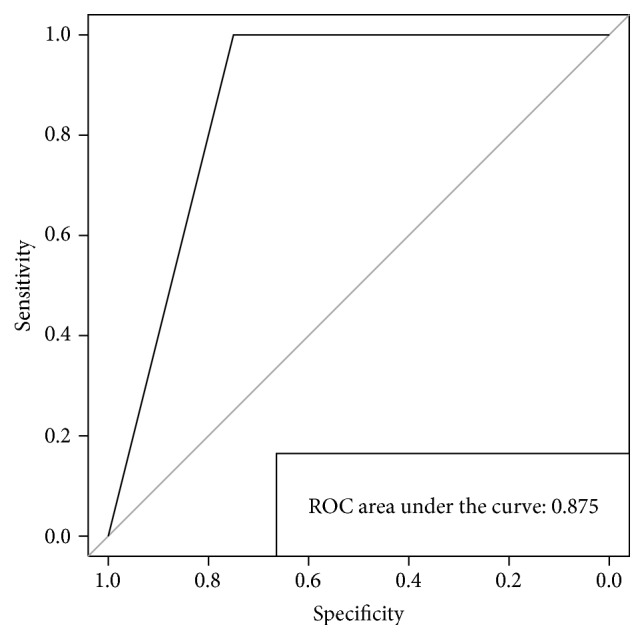
*Receiver Operating Characteristic* (ROC) curve computed by determining the number of true positive (sensitivity) and true negative (specificity) observations when employing the selected features.

The performances are further elucidated by the ROC curve, whose AUC (*area under the curve*) of 0.875 indicates a good capability of the model in assigning specimens to the correct class (Figure 3).

Table 4 lists the parameters applied to the* Support Vector Machines* after the tuning (i.e., parameter optimisation). The computational time of the automatic tuning process is clearly dependent on the range of values to be evaluated and the optimisation method applied for the evaluation. In this case, we optimised the model parameters over a fixed set of default values (see, e.g., [14]) simply by taking the best resulting performance.

**Figure 4:**
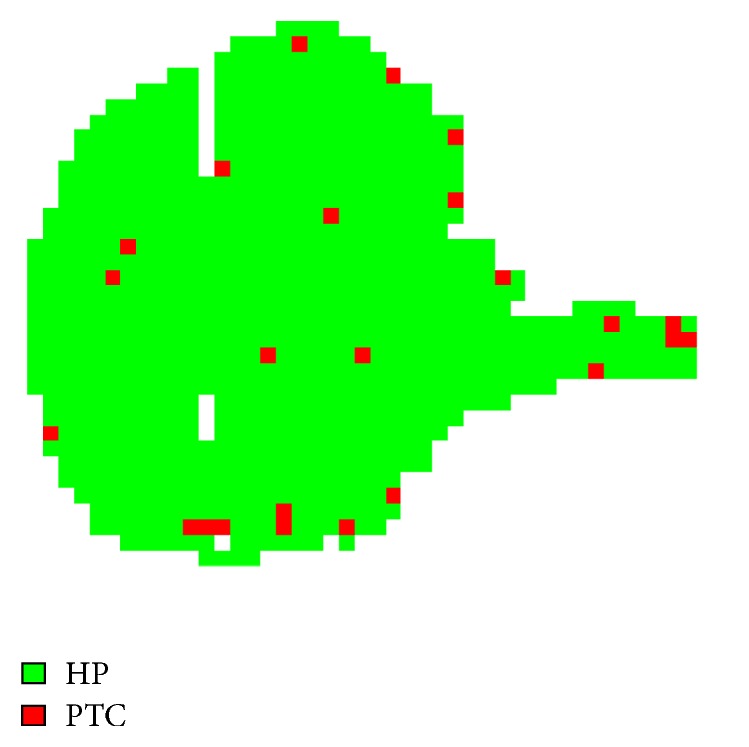
Pixel-by-pixel classification. An entire thyroid cytological smear is displayed. A mass spectrum was acquired for each pixel and the pixel-by-pixel classification has been applied. Green pixels correspond to spectra classified as benign (HP: hyperplastic) while red pixels correspond to malignant (PTC: papillary thyroid carcinoma) spectra.

As described above, MALDI-MSI data is represented by spectra corresponding to pixels of the digitalised tissue image. Instead of performing the classification onto the average proteomic profile only, this operation can be performed onto the individual spectra coming from the single patient as well. In this way, a* spectra-by-spectra* (corresponding to pixel-by-pixel) classification of the patient specimen can be obtained. Since spectra retain their spatial coordinates during the statistical analysis, it is also possible to colour each pixel according to the inferred class (i.e., green for benign and red for malignant). This process resulted in the green and red area picture (Figure 4), providing a tissue area based classification rather than a standard profile classification of the entire proteomic profile.

## 7. Conclusion and Discussion

The work presented here shows the capability of MALDI-MSI to accurately classify unknown specimens obtained from the clinical routine. In this context,* machine learning* techniques (e.g., SVM) may be considered as a valuable approach able to exploit the full potentiality of the MALDI-MSI data, without the need of porting these findings to other clinical tests. This, in turn, allows MALDI-MSI to properly aid the diagnosis of specimens in the daily clinical practice. Importantly, given that MALDI-MSI looks at the sample at the molecular level, the possibility of performing a pixel-by-pixel classification constitutes a key point in the diagnostic process. In fact, areas highlighted by the inference model can represent regions that are undergoing molecular alterations that are not correlated with morphological changes or very tiny groups of cells that escaped the cytomorphological evaluation. Our results clearly suggest broader investigations either on different datasets or on different classification systems (i.e., ensemble classifiers). Moreover, the next studies will evaluate the possibility of MALDI-MSI to provide the information needed for identifying the correct subgroup of the pathology, to assess the disease progression, and to possibly detect the presence of the disease in the very early stages, providing concrete help in diagnoses. Finally, when more data is available, we will also exploit the possibility of classifying tissue specimens providing inference models directly trained on specific localised areas.

## Figures and Tables

**Table 1:** Table listing all the patients enrolled in the study, along with the cytological and histological diagnosis.

Patient number	Cytological diagnosis	Histological diagnosis
Patient #1	THY2	Ben
Patient #2	THY3	Ben
Patient #3	THY4	PTC
Patient #4	THY5	PTC
Patient #5	THY2	Ben
Patient #6	THY5	PTC
Patient #7	THY2	Ben
Patient #8	THY5	PTC
Patient #9	THY3	PTC
Patient #10	THY4	PTC
Patient #11	THY2	Ben
Patient #12	THY4	PTC
Patient #13	THY3	Ben
Patient #14	THY3	PTC
Patient #15	THY4	PTC
Patient #16	THY2	Ben
Patient #17	THY2	Ben
Patient #18	THY3	PTC
Patient #19	THY2	Ben
Patient #20	THY3	Ben
Patient #21	THY4	PTC
Patient #22	THY3	Ben
Patient #23	THY5	PTC
Patient #24	THY2	Ben
Patient #25	THY4	PTC
Patient #26	THY4	PTC
Patient #27	THY2	Ben
Patient #28	THY2	Ben
Patient #29	THY2	Ben
Patient #30	THY5	PTC
Patient #31	THY5	PTC
Patient #32	THY2	Ben
Patient #33	THY5	PTC
Patient #34	THY3	Ben
Patient #35	THY2	Ben
Patient #36	THY3	Ben
Patient #37	THY3	Ben
Patient #38	THY5	PTC
Patient #39	THY5	PTC
Patient #40	THY5	PTC
Patient #41	THY3	Ben
Patient #42	THY4	PTC
Patient #43	THY2	Ben

Ben: benign lesions; PTC: papillary thyroid carcinoma.

**Table 2:** Table displaying the difference in computational time taken by the classification process when employing the feature selection and when not. The tuning parameter grid is the same in both cases.

	Feature selection	No feature selection
RFE	75.656	//
SVM tuning and test	32.392	117.524

Times are displayed in seconds and calculated by the R function *system.time()*.

**Table 3:** Validation performances of the SVM classifier without performing feature selection.

	Accuracy	Sensitivity	Specificity	PPV	NPV	ROC
EV	0.273	0.000	1.000	0.000	0.273	0.500
2x 10-fold CV	0.567	0.000	1.000	0.000	0.567	0.500

In our case, the performances indicate the ability of the algorithm to correctly detect the benignity when the case is filed as THY3.

EV: external validation; CV: cross-validation; PPV: positive predicted value; NPV: negative predictive value.

**Table 4:** Tuning parameters of the *Support Vector Machines*, with and without performing the feature selection. The best parameters are chosen according to the classification performance of the model.

Feature selection	Kernel	Cost	Epsilon	Gamma
RFE	Radial	10	0.1	0.11
No RFE	Radial	10	0.1	1.11

**Table 5:** Validation performances of the SVM classifier after performing the RFE feature selection.

	Accuracy	Sensitivity	Specificity	PPV	NPV	ROC
EV	0.818	0.750	1.000	1.000	0.600	0.875
2x 10-fold CV	0.713	0.625	0.775	0.740	0.767	0.778

In our case, the performances indicate the ability of the algorithm to correctly detect the benignity when the case is filed as THY3.

EV: external validation; CV: cross-validation; PPV: positive predicted value; NPV: negative predictive value.

**Table 6:** Discrepancy between the predicted class and the actual diagnosis.

Sample	Predicted class	True class
Patient #2	Ben	Ben
Patient #9	PTC	PTC
Patient #13	Ben	Ben
Patient #14	PTC	PTC
Patient #18	PTC	PTC
Patient #20	PTC	Ben
Patient #22	Ben	Ben
Patient #34	Ben	Ben
Patient #36	Ben	Ben
Patient #37	Ben	Ben
Patient #41	PTC	Ben
